# Associations among risk perception, health efficacy, and health behaviors for cardiovascular disease: an application of risk perception attitude framework

**DOI:** 10.3389/fcvm.2023.1201789

**Published:** 2023-09-13

**Authors:** Zhiting Guo, Yun Chen, Yuping Zhang, Chuanqi Ding, Mei Li, Linyan Xu, Jingfen Jin

**Affiliations:** ^1^ Nursing Department, The Second Affiliated Hospital of Zhejiang University School of Medicine (SAHZU), Hangzhou, China; ^2^ Public Health Department, Changxing County People’s Hospital, Huzhou, China; ^3^ Emergency Department, Changxing County People’s Hospital, Huzhou, China; ^4^ Nursing Department, Lishui University School of Medicine, Lishui, China

**Keywords:** cardiovascular disease, risk perception attitude framework, health behaviors, risk perception, efficacy belief, primary prevention

## Abstract

**Background:**

There is currently a pervasive prevalence of cardiovascular disease (CVD) risk worldwide and an inadequate amount of action is being taken to promote healthy lifestyle habits. The risk perception attitude (RPA) framework, which classifies individuals based on their risk perception and efficacy belief, enables us to predict their preventive behaviors. We applied the RPA framework to analyze CVD prevention behaviors among Chinese adults and extended its application to CVD objective risk.

**Methods:**

A cross-sectional survey was performed in two sites in Zhejiang Province, from March to August 2022, which contained self-reported CVD risk perception, objective CVD risk, efficacy belief, physical activity, healthy diet, and covariates. We used the RPA framework to categorize participants into four groups, then analysis was conducted to estimate inter-group differences in healthy behaviors. We further conducted a hierarchical logistic regression analysis with individuals' health behaviors as the dependent variable, using three blocks of independent variables.

**Results:**

Among 739 participants, healthy physical activity and healthy diet had significant differences among four RPA groups, *post hoc* tests clarified that the proportion of respondents with healthy PA in the responsive group (61.6%) was significantly higher than that in the other three groups. Risk perception and efficacy belief significantly predicted health behavior against CVD; the relationship between absolute CVD risk and health behavior was moderated by efficacy belief.

**Conclusions:**

Early CVD risk screening is crucial, but tailored support and a proper understanding of personal risk are essential to promote healthy behaviors. Developing communication and behavioral counseling intervention strategies on the basis of the RPA framework has the potential to promote healthy behaviors for CVD prevention.

## Background

Cardiovascular diseases (CVDs) are the leading cause of disease burden in the world, and cardiometabolic, behavioral, environmental, and social risk factors are major drivers of CVDs (1, 2). In China, CVDs were the leading cause of death (36%) (3). Furthermore, metabolic risk factors accounted for the largest proportion of CVD (41.7%), among which the largest risk factor was hypertension (25.0%), followed by high non-HDL-cholesterol (7.8%), abdominal obesity (6.9%), and diabetes (2.0%) (3). That means the CVD burden resulting from modifiable risk factors continues to increase globally. There is a pressing need to focus on controlling metabolic risk factors and improving education to address this severe public health threat.

Lifestyle modification is the foundation of both primary and secondary prevention of CVDs to reduce cardiometabolic risk, which has been emphasized by major evidence-based guidelines (4–6), such as physical activity (PA), healthy diet (HD), and smoking cessation. Despite this, the current status of lifestyle management remains suboptimal (7, 8). Only 39.6% of adults adopt at least three kinds of low-risk lifestyles from the China Nutrition and Health Surveillance (CNHS) (9), likewise, only 11.1% of adults aged 35–75 years follow a healthy diet and only 23.6% have sufficient physical activity according to the China PEACE Million Persons project (10).

Behavior change is difficult and challenging since it requires making changes to deeply ingrained habits and routines. People may experience multiple barriers to adopting healthy lifestyles, including access to care, knowledge, cost, cultural tradition, time constraints, and social pressure (11–13). As we know, the understanding of CVD risk is a prerequisite for adopting a healthy lifestyle and habits (14). Hence, a positive correlation could be found between individual risk perception and health behaviors (15). Risk perception is widely recognized as a dependable indicator of individuals' inclination to adopt precautionary behaviors, as expounded in various theories (e.g., protective motivation theory, health belief model). Nonetheless, the causal connection between risk perception and CVD prevention behavior has been shown to be relatively weak (16–18). On the other hand, people who possess the competency and confidence to manage their own health may be more inclined to believe that adopting a healthy lifestyle can enhance their health status, thus reinforcing their willingness to comply with health behaviors (19). In addition, the propensity to engage in health behaviors will increase as health self-efficacy increases (20). However, there is limited research on the effects of individual perceptions of CVD risk and health self-efficacy on health behaviors among populations with CVD risk.

### Risk perception attitude framework

To understand the relationship between risk perception, health self-efficacy, and health behaviors, we applied the risk perception attitude (RPA) framework in this study. Specifically, the RPA framework proposes that risk perception and efficacy belief not only have a direct impact but also a moderating effect on individuals' health-related behavior (21). According to the RPA framework, people can be categorized into one of four distinct groups based on risk perception and efficacy level. The “Responsive” category refers to individuals who have high levels of both perceived risk and self-efficacy; individuals with a high level of perceived risk but low self-efficacy were labeled as “Avoidant”; individuals with a low level of perceived risk but high self-efficacy were categorized into the “Proactive” group; and individuals with both a low level of perceived risk and self-efficacy were labeled as “Indifferent”. The RPA framework suggests that individuals in the “Proactive” and “Indifferent” categories may not be as likely to actively change their behaviors due to their lower level of perceived risk. However, the “Responsive” group, who have high levels of both perceived risk and self-efficacy, are more likely to be active actors in changing their health behaviors. On the other hand, the “Avoidant” group, who also have high levels of perceived risk but low levels of self-efficacy, may be less likely to take action towards changing their behaviors compared to the “responsive” group. This highlights the importance of considering both risk perception and self-efficacy when understanding health behavior change comprehensively (21).

**Figure 1:**
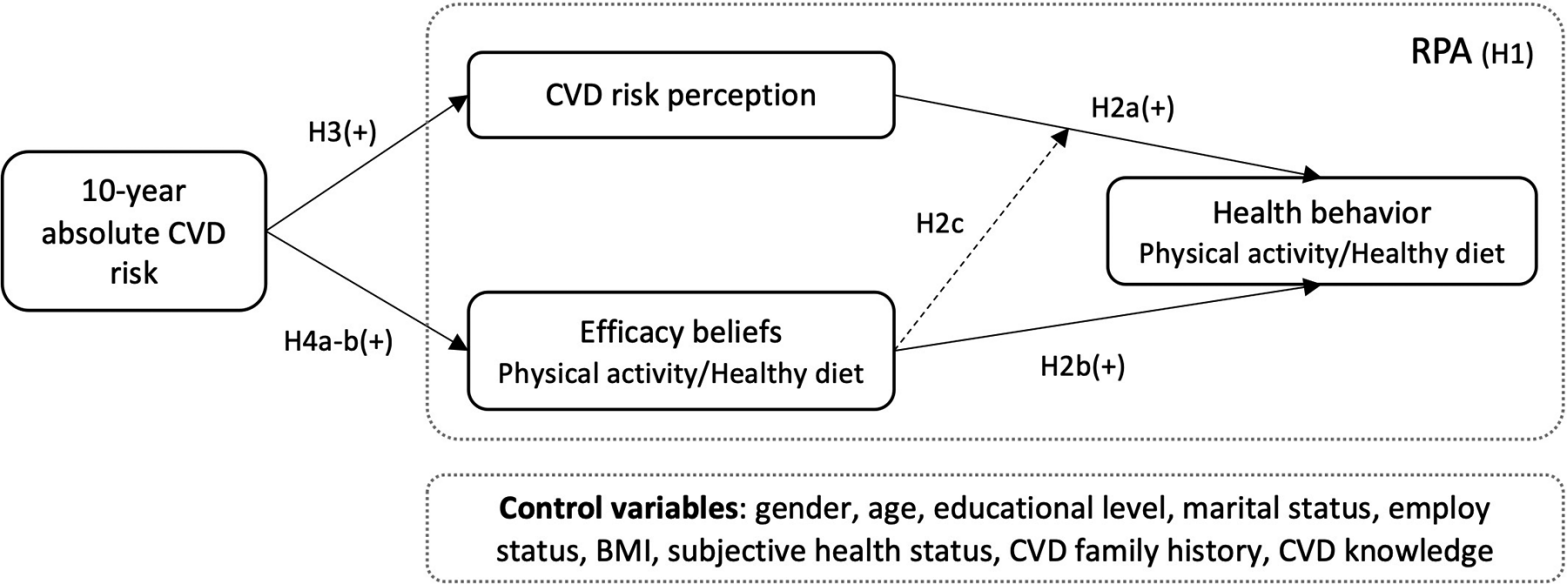
The research model.

Several studies have indeed tested the effectiveness of the RPA framework in promoting health behavior changes related to the prevention of HIV or HPV (22–24), cancer (25, 26), diabetes (27, 28), and COVID-19 (29, 30), as well as pregnancy leisure-time physical activity (31, 32), information seeking (33, 34), safety behavior while driving (35), or using chemical household products (36). However, the RPA framework has yet to be applied to understand health behaviors in response to CVD risk reduction. Furthermore, cultural norms and values can influence how people perceive and respond to risk (37), so it is essential to explore the application of the RPA framework in a non-Western cultural context. The following hypotheses were proposed: (Figure 1)

**H1:** Differences exist among the four RPA categories in health behaviors (physical activity, healthy diet). Participants in “Responsive” and “Proactive” groups will present higher levels of health behaviors (physical activity, healthy diet) than participants in “Indifferent” and “Avoidant” groups. In addition, the four RPA groups differ in their demographic characteristics.
**H2:** Participants’ (a) perceived risk, (b) efficacy belief, and (c) their interaction would positively predict health behaviors (physical activity, healthy diet) toward CVD prevention.

### Cardiovascular disease risk appraisal

Current guidelines for CVD prevention all apply objective absolute estimates of an individual's risk of developing CVD to guide treatment decisions and hierarchical prevention strategies (5, 38, 39). Specifically, lifestyle change was needed for individuals of low risk; moderate-risk individuals should adhere to lifestyle change initially, medication should be considered unless additional risk factors were present or lifestyle modification was inefficacy, and high-risk individuals should accept both blood pressure or cholesterol-lowering medication and lifestyle modification. Risk appraisal provides a platform for healthcare professionals and patients to discuss the absolute CVD risk and potential treatment options, then allows patients to make informed health decisions leading to improved adherence to medication and healthy behaviors (40). CVD risk estimation tools are commonly used to calculate the 10-year or lifetime risk of individuals and directly reflect the impact of multicomponent interventions in large randomized controlled trials (41). However, such hierarchical risk intervention strategies are likely to be effective only if individuals understand their own risks and potential benefits of prevention interventions first, then they are more likely to identify the need for change (42). Several studies reported that mismatch widely occurred between objective calculated CVD risk and individual perceived risk, which in turn affects one's willingness to use or consider preventive medication and adopt healthy lifestyles (15, 43, 44). Some studies reported no correlation found between absolute CVD risk, an individual's perceived risk, and healthy behaviors (16, 45, 46). People with high-perceived CVD risk were more likely to adopt healthy behaviors compared with under-perceived ones, but the effect path was unclear. In addition, there is a limited amount of study to certify the relationship between CVD objective risk, risk perception, efficacy belief, and health behaviors. Examining the prediction role of objective absolute risk on an individual's healthy behaviors, along with their risk perception and efficacy belief, will expand our knowledge of the RPA framework in CVD prevention scenarios. Based on the logical de deduction and literature review, we propose the following hypothesis (Figure 1):

**H3**: 10-year absolute CVD risk is positively associated with CVD risk perception.
**H4**: 10-year absolute CVD risk is positively associated with healthy physical activity efficacy belief (a) and healthy diet efficacy belief (b).

## Methods

### Sampling and procedure

A cross-sectional survey with convenience sampling was conducted in two sites of Zhejiang Province, from March to August 2022. The inclusion criteria were: (1) enrolled as a Zhejiang citizen in the District of Hangzhou or Changxing area; (2) being 20–80 years of age; (3) having no previous diagnosis of CVD; (4) having full medical examination report within at last three months; and (5) being able to read and speak in Mandarin. Participants with critical illness or intellectual disability, or who were pregnant or undergoing treatment for a psychiatric disorder were excluded.

The sample size calculation considers the requirements of regression models and mediating effect analysis, the required sample size should be 10 to 15 times the number of observed variables (47, 48). A total of 9 demographic factors and 7 scale-related dimensions were considered independent variables in our study, assuming an attrition rate of 20%, at least (9 + 7) × 10 × (1 + 20%) = 192.

Data were collected through standardized protocol and questionnaires administered by well-trained researchers. Both a paper-based questionnaire and an online survey platform powered by WJX (www.wjx.com) were provided and anonymized, and participants could choose the survey they preferred. Prior to beginning the questionnaire, a paragraph detailed the research purpose, data usage, and privacy protection. After agreeing to these terms, participants proceeded to the questionnaire page and filled it out independently. The questionnaire included general information, 10-year CVD absolute risk appraisal, CVD risk perception, healthy diet-related efficacy belief, physical activity-related efficacy belief, healthy diet score, and self-reported physical activity. The items were self-assessed by participants except for the 10-year CVD risk appraisal. The 10-year absolute CVD risk was calculated using an online calculator by the investigator after obtaining the indicators from the health check report, with the permission of the participants. If participants were unable to write, the investigator read each item to them, then the questionnaire was completed according to the statements of the participants. The research was approved by the Ethics Committee of the Second Affiliated Hospital of Zhejiang University School of Medicine (No. 2022-0280). After the exclusion of 43 responses with incomplete or invalid answers to the questionnaire, a total of 739 participants were included in the final analysis.

### Measurements

#### General information

Demographic data contained age, gender, height (cm), weight (kg), marital status (coded as married or single), education level (coded as less than junior school, middle school/high school, college school, and above), employment status (coded as employed or unemployed), family history of CVD, and subjective health status. Body mass index (BMI) was determined by dividing weight (kg) by height (m) squared. Family history of CVD was defined as the presence of at least one immediate relative (parent or sibling) with myocardial infarction or stroke (49). Subjective health status was assessed by the response to the question, “In general, how would you rate your health status?” (1 = very poor, 2 = poor, 3 = fair, 4 = good, 5 = excellent) (50).

#### 10-year CVD absolute risk appraisal

The 10-year CVD absolute risk was calculated using the China-PAR (Prediction for Atherosclerotic cardiovascular disease Risk) equation (51) which was developed from the gender-specific Cox proportional hazards model (49). The estimation items included sex, age, geographic region (Northern China/Southern China), urbanization (urban/rural), treated or untreated Systolic blood pressure (mmHg), total cholesterol (mmol/L), high-density lipoprotein cholesterol (HDL-C; mmol/L), currently smoking (yes/no), diabetes (yes/no), waist circumference (WC; cm), and family history of CVD (yes/no). Based on the cut-off value recommended in the Chinese guideline (38), participants were divided into three classes: low risk (<5%), moderate risk (5%–9.9%), and high risk (≥10%). The risk score was obtained through an online calculator (https://www.cvdrisk.com.cn/ASCVD/Eval).

#### CVD risk perception

The Attitude and Beliefs about Cardiovascular Disease (ABCD) Risk Questionnaire (52) was employed to measure people's CVD risk perception, which has confirmed validity in the Chinese population (ABCD-C) (53). The scale contains 26 items of four dimensions: CVD-related knowledge (8 items), risk perception (8 items), perceived benefits and intention to change physical activity (6 items), and perceived benefits and intention to change dietary habits (4 items). For knowledge items, 1 = right answer, 0 = wrong or “I don’t know” answer. Values were summed to a total score, with a higher score indicating a higher level of CVD-related knowledge. Answer options for another three dimensions were presented on a Likert 4-point scale ranging from 1 (strongly disagree) to 4 (strongly agree); a “not applicable” option was added, with a value of 0. Items 15, 21, and 26 were reverse-coded. In the present study, the Cronbach's *α* for the four dimensions was 0.668, 0.936, 0.894, 0.846, respectively.

#### Physical activity and healthy diet efficacy belief

According to social cognitive theory, and following recommendations by Rimal et al. (26), efficacy belief comprises (a) self-efficacy indicating an individual perceived ability to exert personal control, and (b) response efficacy which are the perceived benefits from engaging in a particular behavior (healthy diet, physical activity) (36). For self-efficacy, we used the single item “I am confident that I can exercise at least 150 min a week/ eat at least 500 g per day of fresh fruit and vegetables within the next two months”. For response efficacy, respondents answered the following question: “When I exercise for at least 150 min a week/eat at least 500 g per day of fresh fruit and vegetables, I am doing something good for the health of my heart”. Answer options for both constructs used a 4-point Likert scale (1 = strongly disagree to 4 = strongly agree). These items were extracted from ABCD-C (53). Efficacy beliefs were calculated as the average of self-efficacy and response efficacy. Based on a median split method recommended by Lee et al (36), a high- and a low-efficacy belief group was created.

#### Healthy diet score

A semi-quantitative food frequency questionnaire (FFQ) was used to collect habitual dietary intake through the response to the frequency of eating 18 food types during the past year (54). For each food type, seven frequency classes were presented (“more than 3 times per day”, “1–2 times per day”, “5–6 days per week”, “3–4 days per week”, “1–2 days per week”, “1–3 days per month”, “never or almost never”) (Supplementary Table S1). Based on the recommendations in the updated Chinese Dietary Guideline (55), the healthy diet score was computed based on the weekly intake of six food groups. These groups include fresh fruit, fresh vegetables, whole grains, eating fish and other seafood (more than once per week), beans and bean foods (at least four times per week), and red meat (less than seven times per week). A response that met the criterion scored 1 for each food group, and the total score was summed (full score = 6). The healthy group was defined as those with a total score ≥4 (10, 56).

#### Self-reported physical activity

Self-reported weekly physical activity was assessed through the International Physical Activity Questionnaire-short version (IPAQ) (57). Participants were asked to report the frequency and duration of their engagement in vigorous physical activity, moderate physical activity, walking, and sedentary behavior per week. The IPAQ guidelines for Chinese were used to process and aggregate the responses (58). The test-retest reliability was 0.71–0.93 in the Chinese population, and the criterion-related validity to the accelerometer was 0.60 (59). Participants who engaged in at least 150 min of moderate-intensity physical activities or 75 min of vigorous-intensity physical activities per week were deemed to have met the requirements for sufficient physical activity (38), which was considered healthy in this study.

### Formation of four risk perception attitude groups

The participants were categorized into four RPA groups based on their scores in risk perception, efficacy belief for healthy physical activity, and healthy diet, respectively. We employed the segment approach reported in previous relevant studies (27, 32, 36) to identify RPA groups. A median split of both risk perception and efficacy belief was used to produce four groups, then the groups were classified as high (≥16.82) and low (<16.82) risk perception groups. For efficacy belief, the median value for physical activity and healthy diet was 3.09 and 3.23, respectively; therefore, we split the respondents into high (≥3.09/3.23) and low (<3.09/3.23) efficacy belief groups. Afterward, we created a bi-plot that categorized each risk classification into its respective quadrant: *responsive* (high risk perception + high efficacy), *proactive* (low risk perception + high efficacy), *avoidant* (high risk perception + low efficacy), and *indifferent* (low risk perception + low efficacy).

### Statistical analysis

SPSS 26.0 and the SPSS macro program process plug-in were used to process the data for statistical analysis. The chi-square test and ANOVA were conducted to compare the difference in demographics, risk perception, efficacy belief, healthy physical activity, and healthy diet ratio among four RPA groups. Post hoc analyses used Tukey's HSD or adjusted Bonferroni method. The correlations of the study variables were analyzed by Spearman's correlation. To test the hypotheses, we performed a hierarchical logistic regression analysis with individuals' health behaviors (physical activity and healthy diet, respectively) as the dependent variable. The blocks of indicators were entered into the regression through three steps: (1) the control variables including age, gender, education level, marital status, employment status, BMI, subjective health status, CVD family history, CVD related knowledge; (2) CVD risk perception and efficacy belief; (3) risk perception × efficacy belief. The interaction term was mean-centered to reduce potential multi-collinearity (60). Path analysis was conducted to test the relationships among 10-year absolute CVD risk, CVD risk perception, efficacy belief, and healthy behaviors. Mediating effect tests were conducted following the procedure suggested by Zhonglin Wen et al. (61). *P *< 0.05 was considered statistically significant.

## Results

### Participants’ characteristics among the four RPA groups

The participants were classified into four distinct groups according to their CVD risk perception and healthy PA efficacy belief scores: responsive (*n *= 146), avoidance (*n *= 265), proactive (*n *= 114), and indifference (*n *= 214), as shown in Table 1; similarly, the RPA groups for healthy diet are shown in Table 2. H1 proposed that responsive individuals show the highest level of health behaviors while the indifferent group presented the lowest. To examine H1, we performed Chi-square tests to explore the difference in physical activity and healthy diet among the four RPA groups. The results showed a significant difference in healthy physical activity (*χ*^2^*^ ^*= 141.868, *p *< 0.001) and diet (*χ*^2^*^ ^*= 165.556, *p *< 0.001) across groups. Post hoc tests clarified that the proportion of participants who engaged in healthy physical activity in the responsive group (61.6%) was significantly higher than that in the other three groups, with no significant difference between these three groups. The proportion of participants with a healthy diet in the responsive group (66.1%) was significantly higher than that in the other three groups and the proactive group and avoidance group were significantly higher than the indifference group. Thus, the results were partly consistent with H1.

**Table 1:** Characteristics of the four RPA groups for healthy physical activity (*n *= 739).

Demographics	Responsive (*n *= 146)	Avoidance (*n *= 265)	Proactive (*n *= 114)	Indifference (*n *= 214)	*F*/*χ*^2^, *p-*value
	M ± SD/*n*(%)	M ± SD/*n*(%)	M ± SD/*n*(%)	M ± SD/*n*(%)	
Male (%)	62 (42.5)	108 (40.8)	50 (43.9)	66 (30.8)	8.195, 0.042
Age (years)	54.95 ± 15.56^a^	41.96 ± 13.06^b^	43.32 ± 16.82^b^	36.45 ± 15.06^c^	46.321, < 0.001
Education level					76.478, < 0.001
Junior school or below	46 (31.5)	27 (10.2)	25 (21.9)	21 (9.8)	
Middle/high school	62 (42.5)	86 (32.5)	28 (24.6)	49 (22.9)	
College or above	38 (26.0)	152 (57.4)	61 (53.5)	144 (67.3)	
Marital status					48.664, < 0.001
Single	24 (16.4)	57 (21.5)	30 (26.3)	98 (45.8)	
Married	122 (83.6)	208 (78.5)	84 (73.7)	116 (54.2)	
Employed (%)	51 (34.9)	187 (70.6)	79 (69.3)	156 (72.9)	67.249, < 0.001
BMI (Kg/m^2^)	23.49 ± 3.00^a^	23.33 ± 4.32^a^	22.10 ± 2.63^b^	22.96 ± 4.47^ab^	3.325, 0.019
Subjective health status					25.686, < 0.001
Excellent/good	129 (88.4)	194 (73.2)	104 (91.2)	181 (84.6)	
Fair/poor	17 (11.6)	71 (26.8)	10 (8.8)	33 (15.4)	
CVD family history	11 (7.5)	34 (12.8)	9 (7.9)	14 (6.5)	6.707, 0.082
CVD knowledge	5.82 ± 1.67^a^	5.76 ± 1.88^a^	5.86 ± 2.01^a^	5.65 ± 2.08^a^	0.377, 0.770
10-year CVD risk (%)					79.352, < 0.001
Low risk	54 (37.0)	178 (67.2)	78 (68.4)	172 (80.4)	
Moderate risk	61 (41.8)	61 (23.0)	27 (23.7)	20 (9.3)	
High risk	31 (21.2)	26 (9.8)	9 (7.9)	22 (10.3)	
CVD risk perception	19.85 ± 2.21^a^	20.15 ± 2.33^a^	13.07 ± 2.57^b^	12.64 ± 3.08^b^	479.516, < 0.001
PA efficacy belief	2.77 ± 0.37^a^	3.81 ± 0.24^b^	3.82 ± 0.24^a^	2.59 ± 0.62^c^	395.871, < 0.001
Healthy PA (%)	90 (61.6)^a^	43(16.2)^b^	24(21.1)^b^	23(10.7)^b^	141.868, < 0.001

BMI, body mass index; CVD, cardiovascular diseases; PA, physical activity.

Responsive = high risk perception + high PA efficacy belief; Avoidance = high risk perception + low PA efficacy belief; Proactive = low risk perception + high PA efficacy belief; Indifference = low risk perception + low PA efficacy belief.

Entries sharing the same subscripts are not different at *p *< 0.05.

**Table 2:** Characteristics of the four RPA groups for a healthy diet (*n *= 739).

Demographics	Responsive (*n *= 186)	Avoidance (*n *= 225)	Proactive (*n *= 143)	Indifference (*n *= 185)	*F*/χ^2^, *p-*value
	M ± SD/*n*(%)	M ± SD/*n*(%)	M ± SD/*n*(%)	M ± SD/*n*(%)	
Male (%)	84 (45.2)	86 (38.2)	53 (37.1)	63 (34.1)	5.140, 0.162
Age (years)	52.29 ± 14.94^a^	41.85 ± 13.95^b^	44.04 ± 16.83^bc^	34.82 ± 14.13^d^	43.759, < 0.001
Education level					105.632, < 0.001
Junior school or below	47 (25.3)	26 (11.6)	28 (19.6)	18 (9.7)	
Middle/high school	96 (51.6)	52 (23.1)	41 (28.7)	36 (19.5)	
College or above	43 (23.1)	147 (65.3)	74 (51.7)	131 (70.8)	
Marital status					93.709, < 0.001
Single	30 (16.1)	51 (22.7)	25 (17.5)	103 (55.7)	
Married	156 (83.9)	174 (77.3)	118 (82.5)	82 (44.3)	
Employed (%)	81 (43.5)	157 (69.8)	90 (62.9)	145 (78.4)	53.700, < 0.001
BMI (Kg/m^2^)	23.96 ± 3.65^a^	22.92 ± 4.05^b^	22.48 ± 3.36^bc^	22.81 ± 4.35^bd^	4.664, 0.003
Subjective health status					11.331, 0.010
Excellent/good	142 (76.3)	181 (80.4)	120 (83.9)	165 (89.2)	
Fair/poor	44 (23.7)	44 (19.6)	23 (16.1)	20 (10.8)	
CVD family history	18 (9.6)	27 (12.0)	5 (3.5)	18 (9.7)	7.792, 0.051
CVD knowledge	5.65 ± 1.75^a^	5.89 ± 1.85^a^	5.84 ± 1.92^a^	5.63 ± 2.15^a^	0.936, 0.423
10-year CVD risk (%)					139.821, < 0.001
Low risk	60 (32.3)	172 (76.4)	93 (65.0)	157 (84.9)	
Moderate risk	91 (48.9)	31 (13.8)	29 (20.3)	18 (9.7)	
High risk	35 (18.8)	22 (9.8)	21 (14.7)	10 (5.4)	
CVD risk perception	19.92 ± 2.14^a^	20.14 ± 2.40^a^	13.45 ± 2.63^b^	12.28 ± 3.03^c^	493.710, < 0.001
HD efficacy belief	3.76 ± 0.25^a^	2.91 ± 0.24^b^	3.77 ± 0.25^a^	2.66 ± 0.60^c^	440.945, < 0.001
Healthy diet (%)	123 (66.1)^a^	54(24.0)^b^	44(30.8)^b^	11(5.9)^c^	165.556, < 0.001

BMI, body mass index; CVD, cardiovascular diseases; HD, healthy diet.

* *Responsive = high risk perception high HD efficacy belief; Avoidance = high risk perception + low HD efficacy belief; Proactive = low risk perception + high HD efficacy belief; Indifference = low risk perception + low HD efficacy belief.

Entries sharing the same subscripts are not different at *p *< 0.05.

Also, we examined the differences in demographic characteristics across the RPA groups. The results showed a significant difference except for CVD family history and CVD-related knowledge among RPA groups of healthy physical activity, while a significant difference was also found among RPA groups of healthy diet except for gender, CVD family history, and CVD-related knowledge (Tables 1, 2).

### Correlation analysis

The distribution of 10-year CVD absolute risk, CVD risk perception, PA efficacy belief, and healthy diet efficacy belief was non-normal (Shaprio-Wilk test, *P *< 0.001); additionally, healthy PA and healthy diet data were ordinal in nature. Consequently, Spearman's correlation analysis was performed. The correlations between variables are shown in Table 3, which provides the basis for further testing the hypotheses. Ten-year CVD absolute risk, CVD risk perception, PA efficacy, healthy diet efficacy belief, healthy PA, and healthy diet were in pairwise correlation with each other significantly, except for no relationship being found between CVD risk perception and PA efficacy belief.

**Table 3:** Spearman's correlation analysis (*n *= 739).

	1	2	3	4	5	6
10-year CVD absolute risk	1					
CVD risk perception	0.349**	1				
PA efficacy belief	0.184**	0.033	1			
Healthy diet efficacy belief	0.306**	0.100**	0.470**	1		
Healthy PA	0.147**	0.281**	0.332**	0.146**	1	
Healthy diet	0.183**	0.409**	0.125**	0.367**	0.187**	1

CVD, cardiovascular disease; PA, physical activity.

** *p *< 0.01.

### Verification results of hierarchical regressions

The effects of control variables were examined first (block 1), Subsequently, the main factors containing CVD risk perception and efficacy belief (block 2), the interaction of risk perception and efficacy belief (block 3) were added to test the main and moderating effects. The results of multiple regression for risk perception, efficacy belief and health behaviors are shown in Table 4. We found that risk perception was positively associated with healthy PA (*OR *= 1.247, 95% *CI *= 1.178,1.321), and healthy diet (*OR *= 1.372, 95% *CI *= 1.290,1.460). In addition, the efficacy belief was positively associated with healthy PA (*OR *= 4.734, 95% *CI *= 3.200,7.005), and healthy diet (*OR *= 9.244, 95% *CI *= 5.698,14.995). The results were consistent with H2a and H2b.

**Table 4:** Hierarchical regressions on healthy behaviors (*n *= 739).

	Healthy PA			Healthy diet		
Block 1	*β*	OR (95%*CI*)	Wald	*β*	OR (95%*CI*)	Wald
Age (years)	0.042	1.043 (1.021,1.066)	15.115**	0.031	1.032 (1.011,10.53)	8.843**
Gender (male = 1)	−0.012	0.988 (0.670,1.457)	0.004	0.635	1.888 (1.295,2.752)	10.912**
Education level	0.215	1.240 (0.586,2.622)	0.431	0.039	1.040 (0.514,2.106)	0.012
Marital status (single = 1)	−0.545	0.580 (0.356,0.946)	4.762*	0.724	2.062 (1.296,3.279)	9.338**
Employ status	−0.084	0.919 (0.522,1.618)	0.086	0.609	1.839 (1.060,3.192)	4.695*
BMI (Kg/m^2^)	0.008	1.009 (0.958,1.061)	0.106	0.054	1.056 (1.007,1.107)	5.097*
SHB (poor = 1)	0.625	1.867 (1.120,3.115)	5.726*	0.238	1.269 (0.813,1.980)	1.104
CVD family history	0.239	1.270 (0.690,2.338)	0.591	0.314	1.369 (0.775,2.418)	1.171
CVD knowledge (score)	−0.008	0.992 (0.899,1.094)	0.029	0.016	1.016 (0.926,1.115)	0.117
	${R_{\mathrm{change}}^{2}}_{}$ *_ _*= 0.082, *p *< 0.001			${R_{\mathrm{change}}^{2}}_{}$*_ _*= 0.089, *p *< 0.001		
Block 2						
RP	0.221	1.247 (1.178,1.321)	57.090**	0.316	1.372 (1.290,1.460)	99.921**
PA efficacy belief	1.555	4.734 (3.200,7.005)	60.498**	/		
HD efficacy belief	/			2.224	9.244 (5.698,14.995)	81.177**
*R*^2^	${R_{\mathrm{change}}^{2}}_{}$ =0.232, *p *< 0.001			${R_{\mathrm{change}}^{2}}_{}$*_ _*= 0.346, *p *< 0.001		
Block 3						
RP × PA efficacy belief	0.130	1.138 (1.043,1.242)	8.521**			
RP × HD efficacy belief				0.204	1.226 (1.090,1.380)	11.464**
	${R_{\mathrm{change}}^{2}}_{}$ = 0.012, *p *< 0.001			${R_{\mathrm{change}}^{2}}_{}$*_ _*= 0.014, *p *< 0.001		
Total adjusted *R*^2^		0.326			0.449	

PA, physical activity; BMI, body mass index; SHB, subjective health status; CVD, cardiovascular disease; RP, risk perception; HD, healthy diet; healthy PA final model Hosmer-Lemeshow test, *χ*^2^*^ ^*= 9.591, *p *= 0.295; healthy diet final model Hosmer-Lemeshow test, *χ*^2^*^ ^*= 17.189, *p *= 0.028; *p < 0.05, **p < 0.01.

H2c predicted interaction effects between CVD risk perception and efficacy belief on health behaviors, namely that the relationship among risk perception and health behaviors would strengthen along with efficacy belief. The results revealed an interaction effect exist between risk perception and efficacy belief on healthy PA (*OR *= 1.138, 95% *CI *= 1.043,1.242), as well as healthy diet (*OR *= 1.226, 95% *CI *= 1.090,1.380).

Path analysis by means of regression was used to test the relationships among 10-year absolute CVD risk, CVD risk perception, efficacy belief, and healthy behaviors, so as to verify the hypothesis. The results are shown in Table 5. The findings indicated that only a 10-year CVD risk affects PA efficacy belief positively. The results of multiple regression are shown in the Supplementary Tables S2, S3.

**Table 5:** Hypothesis testing results.

Hypothesis	Pathway	Coefficient	Result
H2a	RP → PA	0.221**	Y
	RP → HD	0.316**	Y
H2b	PA efficacy belief → healthy PA	1.555**	Y
	HD efficacy belief → HD	2.224**	Y
H2c	RP × PA efficacy belief → healthy PA	0.130**	Y
	RP × HD efficacy belief → HD	0.204**	Y
H3	10-year CVD risk → RP	−0.008	N
H4a	10-year CVD risk → PA efficacy belief	−0.137*	Y
H4b	10-year CVD risk → HD efficacy belief	0.110	N

RP, risk perception; PA, physical activity; HD, healthy diet; CVD, cardiovascular disease.

* *p *< 0.05, **p < 0.01; Y, support; N, not support.

#### Mediating effect test

The mediating effect was conducted on the condition of controlling variables. The 95% confidence intervals were obtained by Bootstrap 5,000 times sampling correction (Table 6). The results suggested that 10-year CVD risk predicts a healthy diet, while there was no significance for PA efficacy belief. After the mediating variable HD efficacy belief was included, the predictive effect remained significant. Besides the direct effect of a 10-year CVD risk on healthy diet, the mediating effect of efficacy belief was non-zero, which indicates that a 10-year CVD risk predicts healthy diet not only directly but also indirectly through the mediating effect of efficacy belief. Thus, efficacy belief partially mediates the effect of 10-year CVD risk on a healthy diet. As for healthy PA, after the mediating effect HD efficacy belief was included, the direct effect of 10-year CVD risk on healthy PA decreased while the efficacy belief had a significant positive predictive effect on healthy PA. Therefore, efficacy belief fully mediates the effect of 10-year CVD risk on healthy PA.

**Table 6:** Results of mediation effect analysis.

Model pathways	*c*	*a*	*b*	a*b	a*b 95% Boot CI	*c*′
10-year CVD risk → PA efficacy belief → Healthy PA	−0.057	−0.085*	0.268*	−0.023	−0.075–−0.001	−0.034
10-year CVD risk → DH efficacy belief → Healthy diet	0.157*	0.132*	0.326*	0.043	0.009–0.112	0.114*

* *p *< 0.01.

## Discussion

As we know, this study is the first to investigate the relationship between CVD risk perception, efficacy belief, and healthy behaviors containing healthy physical activity and healthy diet under the RPA framework among the Chinese population at risk for CVD. Consistent with RPA, CVD risk perception and efficacy belief were positively associated with healthy physical activity and healthy diet behaviors. Moreover, an interaction effect between CVD risk perception and efficacy belief in healthy physical activity/healthy diet behaviors was confirmed. In detail, there was a significant association between CVD risk perception and healthy behaviors, but only when the individual had a higher level of efficacy belief. We also verified that the actual health behavior difference was significant among RPA groups, and the responsive group was higher than the other three groups in healthy physical activity and healthy diet. In extending the RPA framework from an individual actual CVD risk perspective, our findings revealed that efficacy belief mediates the predictive effect of 10-year CVD risk on health behavior. This compensation addressed the previously identified lack of individual correlation resulting from the absence of actual CVD risk in a previous study (31). In summary, our findings not only contribute to the current literature on the role of the RPA framework in promoting cardiovascular disease (CVD) prevention behaviors, but also offer valuable insights for developing communication strategies that effectively target the CVD-risk population, particularly adults with a younger age and higher education level, and promote healthy behaviors for CVD prevention.

Efficacy belief of the original RPA framework refers to an individual's confidence and competence to engage in preventive behaviors to avert the threat, as well as their belief in the effectiveness of these behaviors to reduce it (21). The preventive behaviors of CVD include not only lifestyle changes, but also preventive medication adherence and self-health monitoring (38), while individuals may have varying levels of commitment toward different types of behavior depending on their personal values and priorities (62). Thus, we specifically designated the efficacy belief of physical activity and a healthy diet to ensure correct measurement, and then RPA groups on healthy PA and a healthy diet were created respectively. In addition, we classified individuals into four distinct groups based on their CVD risk perception and PA/healthy diet efficacy belief and compared their actual physical activity and healthy diet behavior. The results showed a significant difference in healthy physical activity (*χ*^2^*^ ^*= 141.868, *p *< 0.001) and healthy diet (*χ*^2^*^ ^*= 165.556, *p *< 0.001) across groups. In particular, the responsive group reported the highest ratio of healthy PA and healthy diet, whereas the indifference group reported the lowest. Our findings were consistent with the RPA framework's prediction.

We also observed different profiles of four RPA groups on individuals’ characteristics, such as age, education level, marital status, employment status, BMI, and subjective health status. In particular, the indifference group had the highest proportion of younger, better educated, single, employed, and poor health status, meaning that these individuals were likely to perceive low CVD risk and efficacy belief toward both PA and healthy diet, as well as rarely engaged in healthy behaviors. Those findings suggest that more attention should be focused on young people with CVD risk to improve their understanding of CVD risk and belief in behavior change, through effective risk communication and behavior counseling interventions to promote adherence to a healthy lifestyle (63, 64). However, we did not find significant differences in CVD family history among the four groups. The avoidance group shared the highest proportion of CVD family history indicating that individuals with a family member diagnosed with CVD could heighten their perception of risk, but perceived efficacy was insufficient to motivate them to perform preventive behaviors (65). Some individuals may perceive that their predisposition to certain health conditions is inescapable, and modifying their lifestyle choices may not alter their family history. While there were no significant differences in CVD-related knowledge among the four RPA groups, we found that the indifference group had the lowest level of knowledge as well as the lowest likelihood of engaging in CVD prevention behaviors. These results suggest that increasing CVD-related knowledge may not be sufficient in motivating individuals to engage in preventive behaviors, particularly for those who are indifferent. Our study also highlights the importance of assessing an individual's family CVD history and CVD-related knowledge, that information could provide valuable insight for effective prevention strategies (66).

In examining the core premise of the RPA framework, namely the interaction effect of risk perception and efficacy belief on healthy behavior, we detected a significant interaction effect between risk perception and efficacy belief. That is to say, individuals were more likely to act upon their perceived risk engaging in healthy physical activity and healthy diet against CVD when they perceived higher efficacy, as theorized by Rimal et al. (21). Previous RPA investigations have verified the risk perception/efficacy belief interactions for HIV prevention (24), cancer prevention (25, 26), and self-precaution against COVID-19 (30), although the interaction effect of pregnancy risk perception and leisure-time physical activity efficacy belief was not found, the results also suggested that efficacy belief appeared important in facilitating greater levels of pregnancy leisure-time physical activity (32). In addition, Rains et al. tailored messages for diabetes screenings according to baseline RPA groups, and the results revealed that participants who received matching messages reported greater willingness to engage in self-protective behavior (27). These researches revealed the theoretical and practical value as a guidance to promote self-protective behavior.

We also tested the relationships among 10-year absolute CVD risk, CVD risk perception, efficacy belief, and healthy behaviors, and the results showed that efficacy belief mediated the effect of 10-year CVD risk on healthy PA/diet. However, we did not find a similar mediating effect for risk perception, though they were all positively correlated with each other. These results suggest that early CVD risk screening is crucial, but simply identifying objective risks is not enough. It is equally important to ensure individuals have a proper understanding of their personal risk and are provided tailored support that meets their needs and preferences to encourage healthy behaviors. Particularly, the prevalence of cardiovascular metabolic risk is on the rise for young individuals; providing risk education and behavioral counseling during the early stages of college education or workforce transition can enable young individuals to engage in preventative measures and adopt healthy habits for their overall well-being (31).

### Implications and limitations

#### Implication for future research

Our findings provide some valuable clues for future studies. There is a pervasive prevalence of cardiovascular disease risk worldwide and an inadequate amount of action is being taken to promote healthy lifestyle habits. Our study confirms that risk perception and efficacy beliefs positively influence individuals' health-promoting behavior. Additionally, efficacy beliefs play a significant mediating role in the relationship between objective risk scores and health behaviors. On one hand, the study enhances our understanding of the link between objective CVD risk, risk perception, efficacy beliefs, and health behavior. On the other hand, from the perspective of the RPA framework, this study illuminated specific factors that influence individuals' health behaviors, such as age, education level, and health status, thereby providing valuable insights for further research on the practical application of the theory.

#### Findings from primary care practice

Our research findings have important guidance for the primary prevention practice of CVD. A large number of guidelines have recommended that lifestyle modification is the foundation of CVD prevention to reduce cardiometabolic risk (4–6). However, encouraging the public to engage in self-health management and health promotion is one of the most challenging tasks. Our findings certified that the responsive group had the highest ratio of physical activity meeting the guideline and the healthy diet, and the indifferent group had the lowest. Therefore, primary health care should design communication and behavioral counseling intervention strategies that target the CVD risk population, so as to promote healthy behaviors for preventing CVD. In addition, we reported the characteristics in the indifferent group, and those findings suggest that more attention should be given to young people at CVD risk to improve their understanding of CVD risk and belief in behavior change, through effective interventions to promote adherence to a healthy lifestyle.

#### Limitations

The current study has several limitations. First, the web-based online survey used in our study accounted for the fact that the enrolled sample had a high proportion of young/high-education level respondents, so older participants without smartphones or computers were not included in our study. This may be an important source of potential sample bias. Further study will be conducted to verify the study's conclusion in a broader range of people. In addition, we did not exclude individuals who were unable to write or experienced difficulty in writing, the investigator read each question one by one aloud, and the subjects responded accordingly. The accuracy of the results would be enhanced if the subjects completed the questionnaire themselves, although we designed structured options to improve the accuracy, misclassification of some self-reported psychological measurements is possible and could have led to an overestimation of their attributable effect for behavior. Second, the cross-sectional design may capture only a snapshot of an individual's behavioral and psychosocial status. However, it is possible that the relationship between risk perception, efficacy belief, and healthy behaviors may change over time along with an individual's life circumstances, environmental factors, and exposure to health information. To address this, future research may conduct a longitudinal study with these factors on healthy behaviors at a follow-up stage. Third, although the China-PAR is a well-established and validated CVD risk prediction model in China, it does not include LDL-cholesterol. It is well-known that LDL-cholesterol is an important causal factor for CVD (67). Therefore, future studies could apply alternative risk appraisal models to verify the robustness of the results. Finally, we did not test the RPA framework for all forms of CVD prevention behaviors, such as medication adherence, health monitoring, and stress management. Future research should explore the feasibility and effectiveness of utilizing RPA in these areas to determine whether the framework can be extended beyond other CVD prevention behaviors.

## Conclusion

In the current study, risk perception and efficacy belief have not only main effects but also moderating roles in predicting individuals' behavior against CVD; efficacy belief plays a moderating role in the relationship between actual CVD risk and healthy physical activity and healthy diet. Further, designing communication and behavioral counseling intervention strategies on the basis of the RPA framework has the potential to promote healthy behaviors for preventing CVD.

## Data Availability

The raw data supporting the conclusions of this article will be made available by the authors, without undue reservation.
