# Supplementary material for: Associations among risk perception, health efficacy, and health behaviors for cardiovascular disease: an application of risk perception attitude framework
Source: Front Cardiovasc Med. 2023 Sep 13;10:1201789. doi: 10.3389/fcvm.2023.1201789 (PMC10525708; doi:10.3389/fcvm.2023.1201789)
Supplement: Supplementary file 1 [file Table1.docx]

**Supplementary materials**

**Contents**

**Healthy diet score**

**Regression analysis**

- **Risk perception**
- **Healthy physical activity and healthy diet**

**Healthy diet score**

Food intake frequency during the past year was asked in the questionnaire: ‘daily’, ‘4-6 days per week’, ‘1-3 days per week’, ‘1-3 days per month’, ‘never or almost never’. According to the recommendations of the Chinese Dietary Guidelines[1] and related research[2], we focused on 6 food groups to represent heathy diet contain fresh fruit, fresh vegetable, whole grains (mainly referring to crops except rice and flour, including millet, corn, sorghum, sweet potato, etc.), fish and other seafood (referring to fish, shrimp, crab, shellfish, snails, etc.), bean and bean products (referring to all kinds of soy foods including tofu, dried tofu, beverage made from soybeans, etc.), and red meat (referring to a variety of fresh or processed meat such as pork, beef, lamb, etc.).

**Table S1 The definition of healthy diet score**

| **Food groups** | **‘Healthy’ (score=1)** |
| --- | --- |
| Fresh fruit | every day per week |
| Fresh vegetables | every day per week |
| Whole grains | every day per week |
| Fish and other seafood | ≥ 1 day per week |
| Bean and bean products | ≥ 4 days per week |
| Red meat | <7 days per week |

**Regression analysis**

**Table S2 The results of multiple regression analysis for risk perception(*n*=739)**

|  | Risk perception | | |
| --- | --- | --- | --- |
| Block 1 | *Standard β* | SE | *t* value |
| Age (years) | 0.365 | 0.019 | 5.324^**^ |
| Gender (male=1) | 0.025 | 0.337 | 0.665 |
| Education level | 0.091 | 0.334 | 1.571 |
| Marital status (single=1) | 0.104 | 0.413 | 2.487^*^ |
| Employ status | -0.037 | 0.489 | -0.693 |
| BMI (Kg/m^2^) | 0.075 | 0.043 | 1.962 |
| SHB (poor=1) | 0.084 | 0.425 | 2.293^*^ |
| CVD family history | 0.084 | 0.538 | 2.392^*^ |
| CVD knowledge (score) | 0.037 | 0.087 | 0.972 |
|  | *R^2^_change_*=0.130, Δ*F*=13.279, *p*<0.001 | | |
| Block 2 |  |  |  |
| 10-year CVD risk | -0.008 | 0.381 | -0.135 |
| *R^2^* | *R^2^ _change_* =0.001, Δ*F*=0.018, *p*=0.893 | | |
| Total adjusted *R^2^* |  | 0.131 |  |

**Table S3 The results of multiple regression analysis for healthy behavior(*n*=739)**

|  | PA efficacy belief | | | Healthy diet efficacy belief | | |
| --- | --- | --- | --- | --- | --- | --- |
| Block 1 | *Standard β* | SE | *t* value | *Standard β* | SE | *t* value |
| Age (years) | 0.204 | 0.003 | 3.030^**^ | 0.131 | 0.003 | 1.892 |
| Gender (male=1) | 0.016 | 0.051 | 0.452 | 0.041 | 0.047 | 1.098 |
| Education level | -0.159 | 0.052 | 2.806^**^ | -0.185 | 0.048 | 3.162^**^ |
| Marital status (single=1) | 0.004 | 0.063 | 0.109 | 0.118 | 0.058 | 2.781^**^ |
| Employ status | 0.034 | 0.074 | 0.086 | 0.004 | 0.069 | 0.078 |
| BMI (Kg/m^2^) | 0.019 | 0.007 | 0.515 | 0.027 | 0.006 | 0.711 |
| SHB (poor=1) | -0.241 | 0.065 | 6.726^**^ | -0.021 | 0.060 | 0.573 |
| CVD family history | 0.003 | 0.082 | 0.093 | 0.008 | 0.075 | 0.220 |
| CVD knowledge (score) | 0.201 | 0.013 | 5.434^**^ | 0.152 | 0.012 | 3.990^**^ |
|  | *R^2^_change_*=0.163, Δ*F*=16.984, *p*<0.001 | | | *R^2^_change_*=0.111, Δ*F*=11.290, *p*<0.001 | | |
| Block 2 |  |  |  |  |  |  |
| 10-year CVD risk | -0.137 | 0.058 | 2.329^*^ | 0.110 | 0.053 | 1.817 |
| *R^2^* | *R^2^_change_*=0.006, Δ*F*=5.423, *p*=0.020 | | | *R^2^_change_*=0.004, Δ*F*=3.301, *p*=0.070 | | |
| Total adjusted *R^2^* |  | 0.169 |  |  | 0.115 |  |
